# Mutant p53 gain-of-function stimulates canonical Wnt signaling via PI3K/AKT pathway in colon cancer

**DOI:** 10.1007/s12079-023-00793-4

**Published:** 2023-11-20

**Authors:** Eduardo Alvarado-Ortiz, Elizabeth Ortiz-Sánchez, Miguel Angel Sarabia-Sánchez, Karen Griselda de la Cruz-López, Alejandro García-Carrancá, Martha Robles-Flores

**Affiliations:** 1 Programa de Posgrado en Ciencias Biológicas, Universidad Nacional Autónoma de México, Mexico City, Mexico; 2 Subdirección de Investigación Básica, Instituto Nacional de Cancerología, Secretaría de Salud, Ciudad de México, Mexico City, Mexico; 3 Laboratorio de Virus & Cáncer, Unidad de Investigación Biomédica en Cáncer, Instituto de Investigaciones Biomédicas, Universidad Nacional Autónoma de México & Instituto Nacional de Cancerología, Mexico City, Mexico; 4 Departamento de Bioquímica, Facultad de Medicina, Universidad Nacional Autónoma de México, Mexico City, Mexico

**Keywords:** p53, Canonical Wnt signaling, Mutant p53 gain-of-function, β-Catenin phosphorylation, 5-FU chemoresistance

## Abstract

Aberrant canonical Wnt signaling is a hallmark of colon cancer. The *TP53* tumor suppressor gene is altered in many solid tumors, including colorectal cancer, resulting in mutant versions of p53 (mut-p53) that lose their tumor suppressor capacities and acquire new-oncogenic functions (GOFs) critical for disease progression. Although the mechanisms related to mut-p53 GOF have been explored extensively, the relevance of mut-p53 in the canonical Wnt pathway is not well defined. This work investigated the influence of mut-p53 compared to wt-p53 in β-catenin-dependent Wnt signaling. Using the TCGA public data from Pan-Cancer and the GEPIA2 platform, an in silico analysis of wt-p53 versus mut-p53 genotyped colorectal cancer patients showed that *TP53* (p53) and *CTNNB1* (β-catenin) are significantly overexpressed in colorectal cancer, compared with normal tissue. Using p53 overexpression or p53 knockdown assays of wt-p53 or mut-p53, we found that while wt-p53 antagonizes canonical Wnt signaling, mut-p53 induces the opposite effect, improving the β-catenin-dependent transcriptional activity and colony formation ability of colon cancer cells, which were both decreased by mut-p53 knockdown expression. The mechanism involved in mut-p53-induced activation of canonical Wnt appears to be via AKT-mediated phosphorylation of Ser 552 of β-catenin, which is known to stabilize and enhance its transcriptional activity. We also found that while wt-p53 expression contributes to 5-FU sensitivity in colon cancer cells, the RITA p53 reactivating molecule counteracted the resistance against 5-FU in cells expressing mut-p53. Our results indicate that mut-p53 GOF acts as a positive regulator of canonical Wnt signaling and participates in the induction of resistance to 5-FU in colon cancer cells.

**Graphical abstract:**

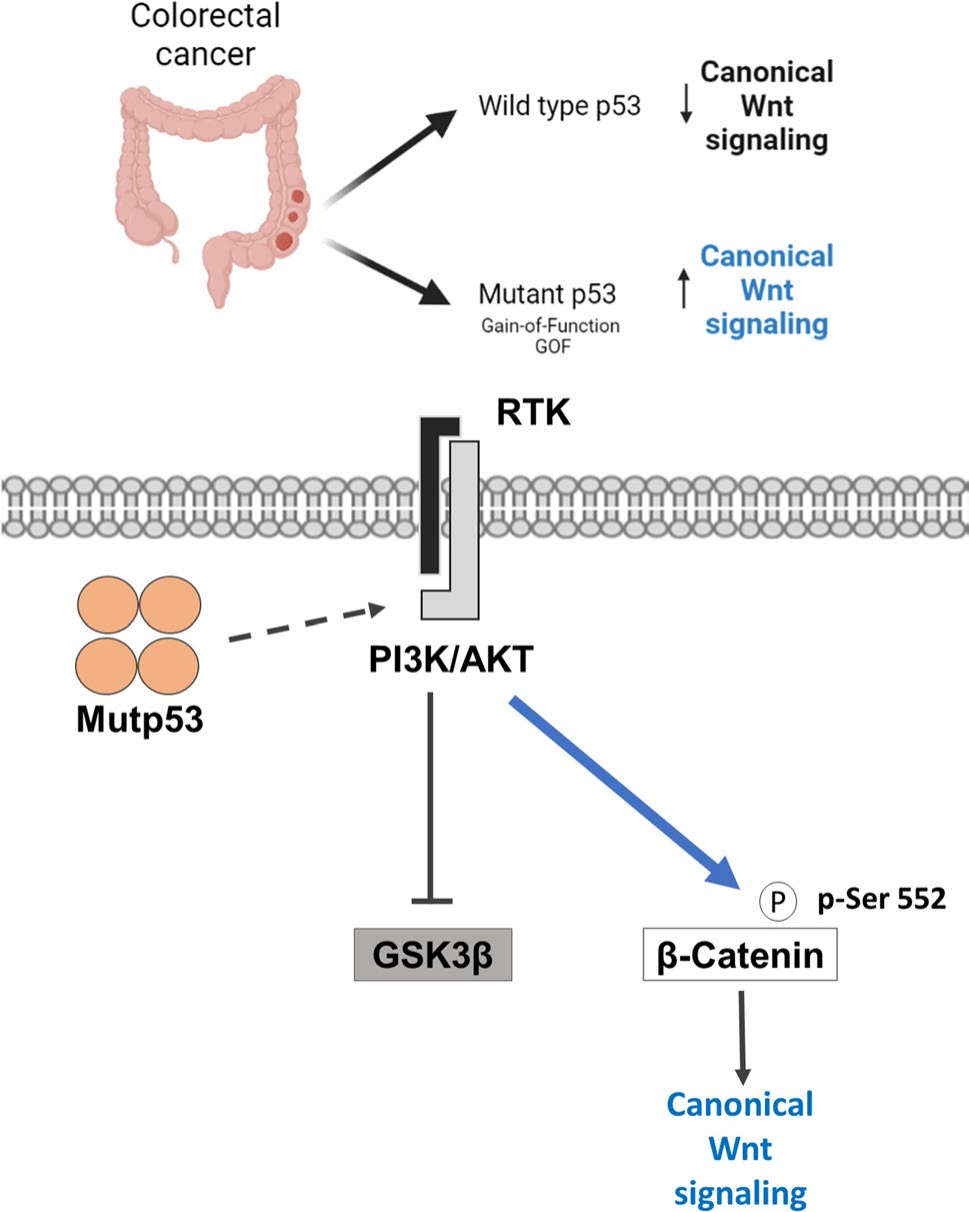

## Introduction

An aberrant canonical Wnt pathway is one of the major driving forces of the initiation and progression of colorectal cancers (Clevers and Nusse 2012). Much experimental evidence has demonstrated that mutations in the Adenomatous Polyposis Coli (*APC*) gene, an essential negative modulator of β-catenin-dependent canonical Wnt signaling, trigger colon cancer progression. However, other genetic alterations are frequently observed in the intestinal and colorectal epithelium, including *K-RAS*, *SMAD4*, and *TP53*, which are closely related to cancer development (Fearon and Vogelstein 1990; Vogelstein et al. 2013).

Known as the “*guardian of the genome*,” p53 is an important tumor suppressor that is altered in more than 50% of solid tumors, including colorectal cancer (Alvarado-Ortiz et al. 2021; Mantovani et al. 2019). Activities driven by p53 include the induction of cell-cycle arrest, expression of pro-apoptotic genes, and response to DNA damage, among others (Vousden and Ryan 2009). The main alterations in the *TP53* gene include mutations in hotspot sites, which are observed in the DNA binding domain (DBD), for example, R175H, R273H, or R248Q sites, while alterations in transactivation or tetramerization domains are less frequent. A consequence of alterations in the DBD of p53 (mut-p53) is the loss of its tumor suppressor capacities and its canonical functions and increases multiple processes related to malignancy (Kim and Lozano 2018). These oncogenic effects associated with mut-p53 are termed gain-of-functions (GOFs) and drive processes related to tumorigenesis, migratory and metastatic functions, metabolic reprogramming, immune evasion, and redox disbalance, among many other mechanisms (Alvarado-Ortiz et al. 2021).

In this work, we investigated the role of mut-p53 expressed by colon cancer cell lines in the canonical Wnt pathway. We show that mut-p53 increases β-catenin-dependent transcriptional activity through the PI3K/AKT axis. Moreover, our findings suggest that the mut-p53 GOF participates in the induction of chemoresistance to 5-FU in colon cancer cells.

## Materials and methods

### Reagents and antibodies

The following primary antibodies used in the present work were obtained from Santa Cruz Biotechnology (Dallas, TX, USA): p53 DO1 (Cat. No. sc-126), p-AKT1/2/3 ser-473 (Cat. No. sc-7965), total AKT (Cat. No. sc-1618), p-GSK-3β (Cat. No. sc-373800), c-myc (Cat. No.sc-40), GAPDH (Cat. No. sc-48167), and MDM2 (Cat. No. sc-13161). Non-phospho (active) β-catenin (Cat. No. 8814) and phospho-β-catenin (Ser552) (Cat. No. 9566) were purchased from Cell Signaling Technology (Danvers, MA, USA). β-catenin (Cat. No.14-2567-82) was from eBioscience (Santa Clara, California, USA), and p21WAF1/Clip1 (Cat. No. SX118) was obtained from Dako (Santa Clara, California, USA). The secondary antibodies used were goat anti-IgG-HRP conjugated (Cat. No. sc-2320), rabbit anti-IgG-HRP conjugated (Cat. No. sc-2013), and mouse anti-IgG-HRP conjugated (Cat. No. sc-2005), obtained from Santa Cruz Biotechnology (Dallas, TX, USA). The PI3K/AKT inhibitor wortmannin was obtained from Sigma (Cat. No. 19545-26-7), and the p53 Activator III compound RITA was from Santa Cruz Biotechnology (Cat. No. sc-202743).

### In silico analysis

A comparative analysis of the expression levels of *TP53*, *CTNNB1*, *CDKN1A*, and *MYC* was performed using Gene Expression Profiling Interactive Analysis (GEPIA) from colon adenocarcinoma (COAD) patients versus *The Cancer Genome Atlas* (TCGA) normal + Genotype-Tissue Expression (TEx) normal. The analysis included 275 tumoral and 349 nontumoral patient samples (Tang et al. 2017). In the case of genotyped patients with altered p53 (mutp53) and unaltered p53 (wtp53), they were subclassified starting from data obtained from *TCGA* using colon adenocarcinoma samples (Pancancer, n = 594) (Hoadley et al. 2018). The analysis was performed with cbioportal.com.

### Cell culture

All cells were obtained from American Type Culture Collection (ATCC) (Manassas, VA, USA) and authenticated by STR DNA profiling analysis in June 2018 at the Instituto Nacional de Medicina Genómica (INMEGEN) in Mexico City. The different genetic contexts of the cancer cell lines used in this work were the following: the human RKO malignant cells are the prototype of BRAF-driven colon cancer cells that express wild-type p53, and wild-type APC exhibit highly inducible canonical Wnt signaling with no basal activation (Ahmed et al. 2013). The human carcinoma SW480 and SW620 cell lines are the prototypes of KRAS-driven cancer cells. They express mutated KRAS and p53 proteins (*KRAS* G12V and *TP53* R273H, respectively) and display constitutively active canonical Wnt signaling because they express a truncated APC protein (Ahmed et al. 2013). RKO cells were cultured in Dulbecco’s modified Eagle’s medium (DMEM) supplemented with 10% Fetal Bovine Serum (FBS), antibiotics (200 mg/ml Streptomycin and 120 mg/ml Penicillin), and 2 mM L-glutamine. SW480 and SW620 cells were maintained in DMEM F-12 supplemented with 10% FBS and 2 mM glutamine. The non-small-cell lung cancer cell line H1299 (ATCC® CRL-5803™), which is null for p53, was used and maintained in RPMI (Thermo Fisher Scientific, Inc., USA) with 10% Fetal Bovine Serum (FBS) (Giaccone et al. 1992.). The cells were incubated in a humidified atmosphere at 37 °C containing 5% CO_2_.

### Spheroid culture (3D cultures)

The H1299 cancer cell line was used to grow spheres in MammoCult™ medium (Stem Cell Technologies®) under the serum-free conditions specified by the supplier with heparin and antibiotics. The cell density was 8000 cells/mL in a volume of 8 mL in a 100 mm ultra-low attachment culture dish. Spheres were grown for 72 h in a humidified atmosphere at 37 °C containing 5% CO_2_.

### Transfection assays

Knockdown of p53 was performed using the SMARTpool siGENOME platform. Lipofectamine 2000 (Invitrogen) and Opti-MEM (Gibco) were used according to the manufacturers’ protocols. An amount of 25–100 nM of SMARTpool siGENOME p53 siRNA (M-993329-03-0010) and siGENOME Nontargeting #1 (D-001210-01-05) were used in the RKO and SW480 cell lines. In the case of transient transfections of wtp53 and its mutants into H1299 cells, they were seeded on 35 mm culture dishes, and 5 μg were used for each condition: pCMV (Empty vector), pCMV WTp53 (wtp53), pCMV R175H (R175H p53), or pCMV R273H (R273H p53). After 24 h, the medium was changed, and lysates were obtained 72 h after transfection.

### TOP reporter gene assay

Luciferase reporter gene assays were performed to measure β-catenin-dependent transcriptional activity. The cells were co-transfected with 1 μg of the reporter plasmid (M50 Super 8× TOPFlash) or control plasmid (Super 8× FOPflash) with 10 ng of the pRL luciferase plasmid (transfection control) and with scramble or siRNA p53. The reporter activity in the cell lysates was measured 72 h after transfection with the Dual-Luciferase Assay kit (Promega, Madison, WI, USA). The activity units were normalized to Renilla luciferase activity.

### Lentiviral transduction

SW480 cancer cells were transduced with control shRNA (sc-108080) or shp53 (sc-29435-V) lentiviral particles according to the manufacturer’s protocol. Then, cells were selected with puromycin (3 ug/mL) for 2 weeks. After that, cells were validated and used for posterior analysis.

### Western blotting

The cells were lysed in ice-cold RIPA buffer (50 mM Tris–HCl pH 8, 150 mM NaCl, 1% NP40, 1 mM EDTA, 0.1% SDS, 0.5% Sodium deoxycholate, 1 mM Phenylmethylsulfonyl fluoride (PMSF), 10 μg/mL leupeptin, and 0.1 mg/mL trypsin inhibitor. Then, the protein in cell extracts was quantified by Bradford assay. Protein samples (15–30 μg) were separated by 10% SDS-PAGE and transferred to nitrocellulose membranes (Bio-Rad). Membranes were blocked with 5% fat-free milk in TBS at room temperature for one hour. The primary antibody was incubated overnight at 4ºC and then with the secondary antibody at room temperature for 2 h. Detection was performed using the SuperSignal Kit (Pierce) in a C-DiGit Blot scanner (LI-COR Bioscience, Lincoln, NE, USA), and analysis was performed using Image Studio™ Lite Software (LI-COR Biosciences).

### Immunoprecipitation assay

SW480 cells treated in the absence or the presence of RITA (1 μM) for 72 h were washed with PBS and lysed using RIPA lysis buffer supplemented with protease and phosphatase inhibitors. For reciprocal immunoprecipitation of MDM2 and mutp53, the cell lysate was adjusted to 1 mg/mL and precleared with 10 μl protein A/G agarose beads for 30 min. Then, the lysate was incubated with 1 μg/mL primary antibody at 4 °C for 1 h with gentle shaking. The complexes were immunoprecipitated overnight with 15 μl protein A/G agarose beads (30%). The complexes were washed four times with PBS 1X supplemented with Triton X-100, trypsin inhibitor, PMSF and protease and phosphatase inhibitors.

### Colony formation assay

Scramble or p53-silenced cancer cells were seeded at 100 cells/well in p96-well culture plaques with 100 μl cell culture medium supplemented with puromycin (3 μg/ml). Cells were incubated for 11 days, and the medium was changed every 3 days. Then, cells were fixed with absolute methanol and stained with crystal violet (0.5%) dissolved in 25% methanol. Colony formation efficiency was measured by quantifying the relative area of stained colonies using ImageJ.

### Cell viability assay

The MTT assay was performed to measure the effect of 5-FU (5-fluorouracil) on the cell viability of colon cancer cells. RKO, SW480, or SW620 cells were seeded in 96-well plates at 2 × 10^4^ cells per well. After 24 h, cells were treated with different doses of 5-FU ranging from 1 to 2500 μM for 72 h. In the case of RITA treatment, 1 μM was used for co-treatment with 5-FU. Then, the medium was removed and the cells were incubated with 0.5 mg/mL MTT for 3 h at 37 °C. After that, formazan crystals were dissolved with acidified isopropanol (pH = 4), and the absorbance was measured at 570 nm.

### Statistical analysis

Data are expressed as means ± standard error of the mean (SEM) from at least three independent cell preparations. The analysis was performed by one-way ANOVA followed by Dunnet post hoc test, while a Student’s t-test was employed to compare the means of two independent groups. Because the samples of in silico analysis do not show a normal distribution, the Mann–Whitney U test was considered to compare two independent groups (wtp53 vs. mutant p53).

## Results

### Colorectal cancer patients with mutant p53 overexpress β-catenin and the canonical Wnt target gene *MYC*

**Fig. 1:**
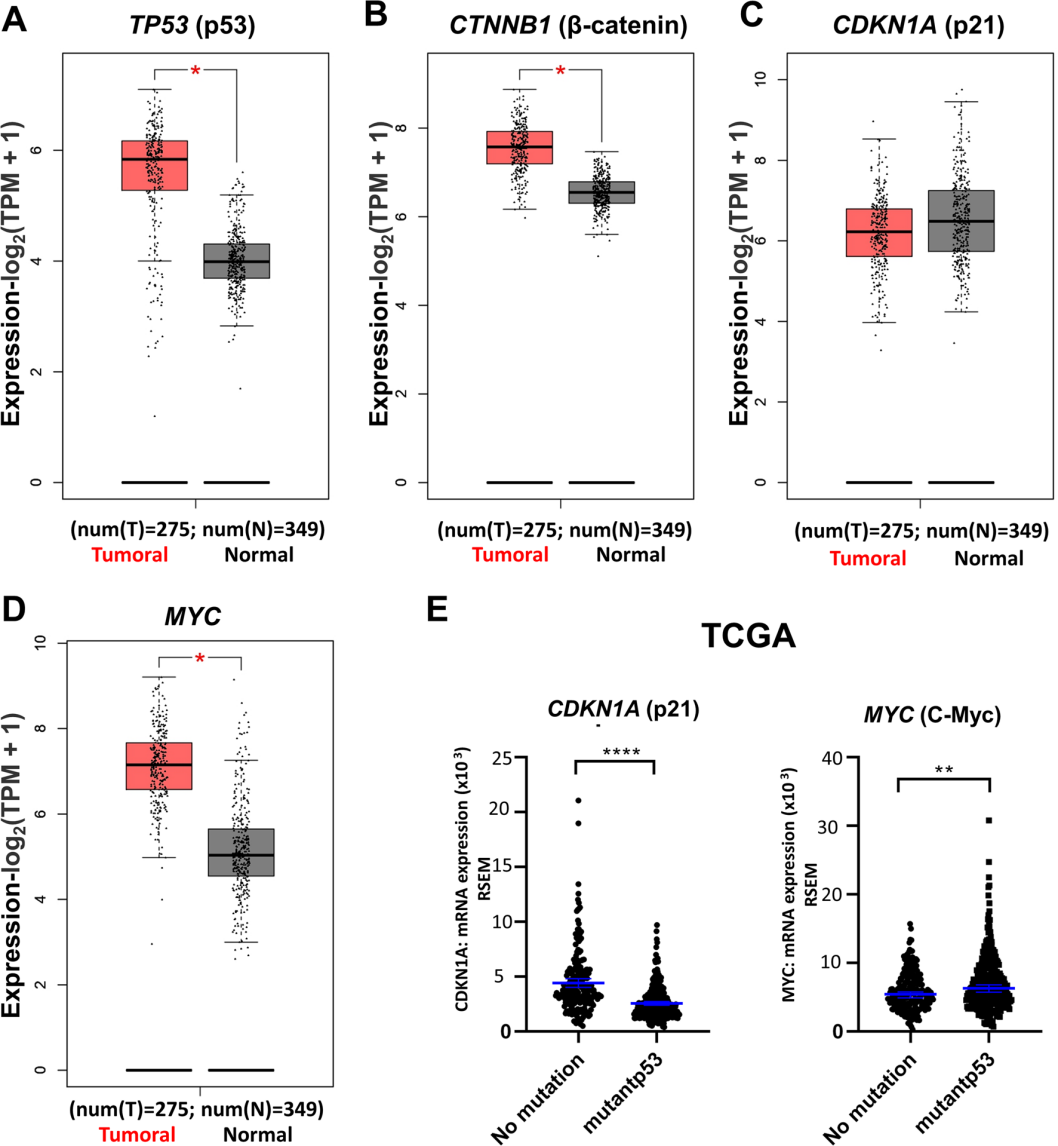
Patients with p53 alterations in colon adenocarcinoma samples show high expression of MYC, a canonical Wnt target. **A**–**C** Colon adenocarcinoma samples from patients show increased levels of *TP53* and *CTNNB1* but not *CDKN1A* in the GEPIA database. **D** Expression of canonical Wnt target (Myc) in normal and tumoral colon adenocarcinoma samples obtained from the GEPIA database. E. Expression of *CDKN1A* (p21) and *MYC* (c-myc) in tumoral samples classified by p53 status (wild-type and mutant) in data obtained from TCGA-Pan Cancer. Graphs represent *RNA-Seq* by expectation–maximization (RSEM). Nonparametric analysis was used for TCGA data, and the graph represents the median with the 95% confidence interval, **p < 0.01

*TP53* is one of the most frequently altered suppressor genes observed in solid tumors. As the first approach to investigating the possible correlation between altered *TP53* gene expression and canonical Wnt signaling in colorectal cancer patients, an in silico analysis was performed using the TCGA public database (Fig. 1A). To accomplish a comparison of colorectal cancer tissues with normal ones, we used the GEPIA platform to make comparisons of mRNA expression levels of *TP53* (p53), *CTNNB1* (β-catenin), and a typical target gene of p53, *CDKN1A* (p21). As shown in Fig. 1, *TP53* and *CTNNB1* were found to be significantly overexpressed in colorectal cancer compared to normal tissue (Fig. 1A, B). This effect was not observed with *CDKN1A* (p21), consistent with the alteration and nonfunctionality of wtp53 in this type of cancer (Fig. 1C). Comparing the expression of c-Myc, a target gene of the canonical Wnt pathway, in wtp53 versus mut-p53 colorectal samples, it was clearly shown that those patients with altered p53 display higher levels of c-Myc expression (Fig. 1D), which was also observed in the TCGA Pancancer database, in which a higher expression of c-Myc was also found in colorectal cancer patients expressing mut-p53 compared to wt-p53 expression (Fig. 1E). Interestingly, the expression of *CDKN1A* was higher in those tissues with endogenous wtp53 (Fig. 1E). These results suggest, therefore, that canonical β-catenin Wnt signaling may be more active in mutp53-expressing patients and that it may be related to TP53 status in colon cancer cells.

### The *TP53*-null H1299 cells transfected with wt-p53 or mut-p53 show that only wt-p53 diminishes β-catenin levels

**Fig. 2:**
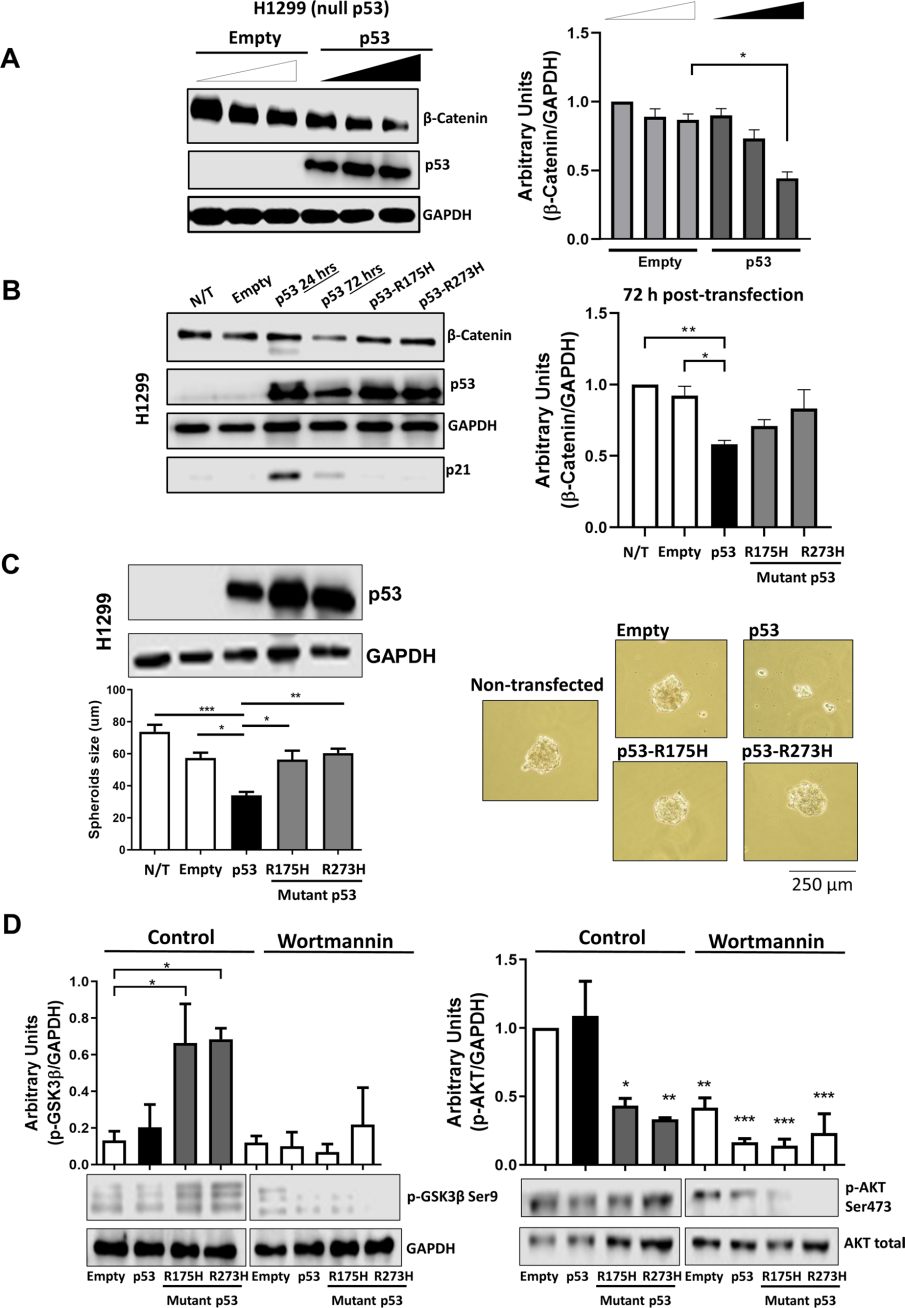
Mutant p53 gain-of-function acts as a positive regulator of canonical Wnt signaling, while wild-type p53 version represses it in the H1299 cell line. **A** Graph and representative immunoblot of β-catenin corresponding to the H1299 cell line (null to *TP53*) transfected with different doses of wtp53 or empty vector; GAPDH was used as a loading control. **B** Graph and representative immunoblots of β-catenin, p53, and p21 obtained from overexpression assays of cells transfected with empty vector, wtp53, R175H p53, and R273H p53 for 72 h. **C** Immunoblot of p53 and comparation of the sizes of spheres obtained from the H1299 cancer cell line transfected with empty vector, wtp53, R175H p53, and R273H p53. Representative spheres were grown for 72 h. **D** Representative immunoblots corresponding to p-GSK3β Ser9 and p-AKT Ser473 from transfected H1299 spheres and treated with either wortmannin inhibitor (150 nM) or DMSO control; GAPDH and total AKT were used as loading controls. Densitometric analysis from at least three independent experiments is shown in graphs, (means ± SEM). *p < 0.05, **p < 0.01, ***p < 0.001

To investigate the specific effects of wt-p53 compared with mut-p53 in Wnt/β-catenin signaling, we first employed as a model the *TP53*-null H1299 cell line to ectopically express wtp-53 or the mutated versions R175H-p53 and R273H-p53. We transfected increasing concentrations of wt-p53 or empty vector and examined its effect on total β-catenin levels. The results presented in Fig. 2A show that wt-p53 was able to negatively affect β-catenin expression levels in a dose-dependent manner. When we transfected the mutated versions of p53 or wt-p53 to compare their effects on β-catenin levels, we observed again that only wt-p53, but not its mutants, diminished β-catenin levels at 72 h post-transfection (Fig. 2B). We only detected the expression of p21, a typical wt-p53 gene target, in cells expressing wt-p53, corroborating the specific effects of wt-p53 (Fig. 2B). Interestingly, when we compared the protein levels of wtp53 and mutp53, 24 h and 72 h after transfection, our results suggested that the mutant versions of p53 are more stable than wt-p53 (Fig. 2B), but most importantly, after 72 h, β-catenin levels were not increased as a result of mut-p53-R273H expression (the same version of mut-p53 expressed in colon cancer cells) with respect to the levels found in nontransfected or empty controls.

Then, we explored the effects of p53 and its mutants on sphere-forming ability in H1299 spheroid cultures. In this context, *TP53*-null H1299 cells were transfected with wt-p53 or mut-p53 and cultured in ultra-low-adherence plates for 72 h as described in the Methods section. Nontransfected cells were considered as controls. The expression of wt-p53 and mut-p53 at 72 h after transfection were validated by western blotting, as can be seen in Fig. 2C. We observed that spheroids expressing wt-p53 were significantly smaller than those formed in cells transfected with empty vector or with mut-p53 versions (Fig. 2C), indicating that wild-type p53 negatively affects cell proliferation. It has been reported that mut-p53 may stimulate proliferation in other cancer types (breast and prostate cancer) by activating the PI3K axis (Muller et al. 2009; Valentino et al. 2017). In turn, PI3K/Akt can stimulate canonical Wnt signaling by inducing the inhibition of GSK-3β via its phosphorylation at Ser 9. To explore the mechanism by which wt-p53 versus mut-p53 affect H1299 spheroid proliferation, H1299-transfected spheroids were treated in the absence or the presence of the PI3K inhibitor wortmannin, and the effect on GSK-3β phosphorylation status at Ser 9 (inactivation), or the Akt phosphorylation status at Ser 473 (activation), was examined by western blotting. The results shown in Fig. 2D indicate that in the absence of wortmannin, mut-p53 favored a GOF that increased p-GSK-3β Ser 9 levels, and therefore may induce activation of canonical Wnt by stabilizing β-catenin levels, while wt-p53 or empty vector did not show this effect. Interestingly, we observed that wortmannin treatment not only greatly reduced GSK-3β inactivation but also induced a significant reduction in p-AKT Ser 473, which resulted in greater inhibition under conditions where mutant versions of p53, particularly the mut-p53-R273H version, were expressed, indicating that mut-p53 GOF activates the PI3K/AKT axis (Fig. 2D).

### Mutant p53 stimulates the Wnt/β-catenin pathway in colon cancer cells

**Fig. 3:**
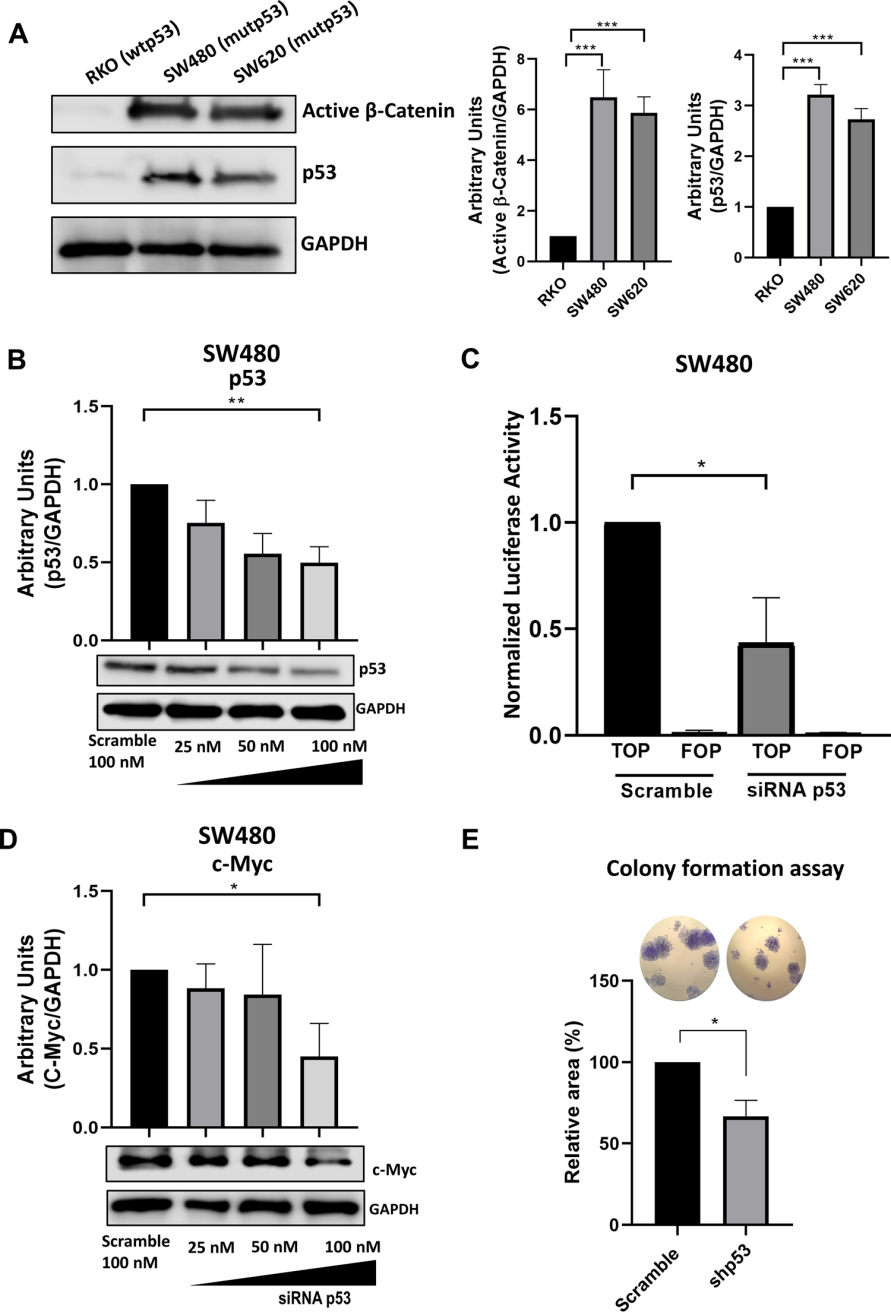
Mutant p53 knockdown in the SW480 cell line decreases canonical Wnt pathway. **A** Comparation of non-phosphorylated β-catenin (active) and p53 protein levels measured in different colon cancer cell lines with different *TP53* status, RKO (wild-type p53), SW480 (R273H p53), and SW620 (R273H p53). Densitometric analysis of immunoblots of p53 and active β-catenin was performed using GAPDH as a loading control. **B** Representative immunoblots and graph of p53 protein in SW480 under different doses of siRNA p53 (25–100 nM); the scramble condition was used as a control (100 nM). **C** Luciferase activity measured with the TOP/FOP system in both scramble and siRNA p53 (100 nM) conditions. The relative units were normalized to Renilla activity. **D** Representative immunoblot and graph of c-myc normalized to GAPDH. E. Representative colonies and relative area quantification corresponding to scramble and shp53 conditions of SW480-transduced cells. Graphs represent densitometric analysis from at least three independent experiments (means ± SEM). *p < 0.05, **p < 0.01, ***p < 0.001

Once we knew in a null-*TP53* cell context model that mut-p53 induces β-catenin stabilization levels and confirmed that wt-p53 induces the opposite, negatively regulating the canonical Wnt pathway, we focused on investigating the role played by mut-p53 on the canonical Wnt pathway in colon cancer cells. As described in detail in the Materials and Methods section, we used RKO cells expressing wild-type p53 and wild-type APC (normal Wnt activation) as well as SW480 or SW620 cells expressing mut-p53 (*TP53* R273H) with constitutively active canonical Wnt signaling because they express a truncated version of APC. Consistent with this, the analysis of the active (non-phosphorylated) β-catenin levels found in SW480 and SW620 by western blotting were much higher in these cells compared with those found in RKO cells, as can be observed in Fig. 3A. Interestingly, it can also be observed in this Figure that endogenous mut-p53 expression levels are much higher in SW480 and SW620 cells than the wt-p53 levels expressed in RKO cells. We then blocked the mut-p53 expression by transient knockdown with siRNA and evaluated the knockdown efficiency using western blotting. Figure 3B shows that expression of mut-p53 was decreased considerably in SW480 cells transfected with the siRNA plasmid with respect to those transfected with the scramble control plasmid (Fig. 3B). Then, we analyzed the effect of mut-p53 knockdown on transcriptional activity mediated by β-catenin in SW480 cells. SW480 cells were co-transfected with siRNA p53 and luciferase reporter plasmid pTOP or with the reporter control plasmid pFOP. The β-catenin-dependent transcriptional activity was measured 72 h after co-transfection. The results in Fig. 3C clearly show that mut-p53 knockdown decreased β-catenin transcriptional activity. Consistent with this, increasing the concentration of siRNA mut-p53 blocked the expression of c-myc, a typical canonical Wnt gene target, in a dose-dependent manner (Fig. 3D). To study the effect of mut-p53 knockdown in a functional role, we performed a colony formation assay in scramble- or shp53-transduced SW480 cancer cells. In line with findings that silencing mutant p53 diminishes canonical Wnt pathway activation, we observed a significantly decreased capacity of mut-p53-silenced cells to form colonies compared to control (scramble) cells (Fig. 3E). Altogether, these results indicated that mut-p53 stimulates canonical Wnt signaling in colon cancer cells.

### Mut-p53 stimulates canonical Wnt signaling through Akt-mediated β-catenin Ser 552 phosphorylation

Because colon cancer cells co-expressing mut-p53 and truncated version of APC, such as SW480 cells, do not possess a functional β-catenin degradation complex, we reasoned that the inhibition of GSK-3β by its phosphorylation at Ser 9 observed in a null-p53 H1299 cell line expressing mut-p53 cannot affect the β-catenin levels in colon cancer SW480 cells, and thus, another mechanism must exist in these cells to explain how mutant p53 can stimulate β-catenin-mediated transcriptional activity. In this regard, it has been demonstrated in HCT116 colon cancer cells and in intestinal organoid cultures that phosphorylation of β-catenin at Ser 552 by AKT contributes to β-catenin stability and enhances its transcriptional activity (Behrouj et al. 2021; Wang et al. 2020; Fang et al. 2007).

**Fig. 4:**
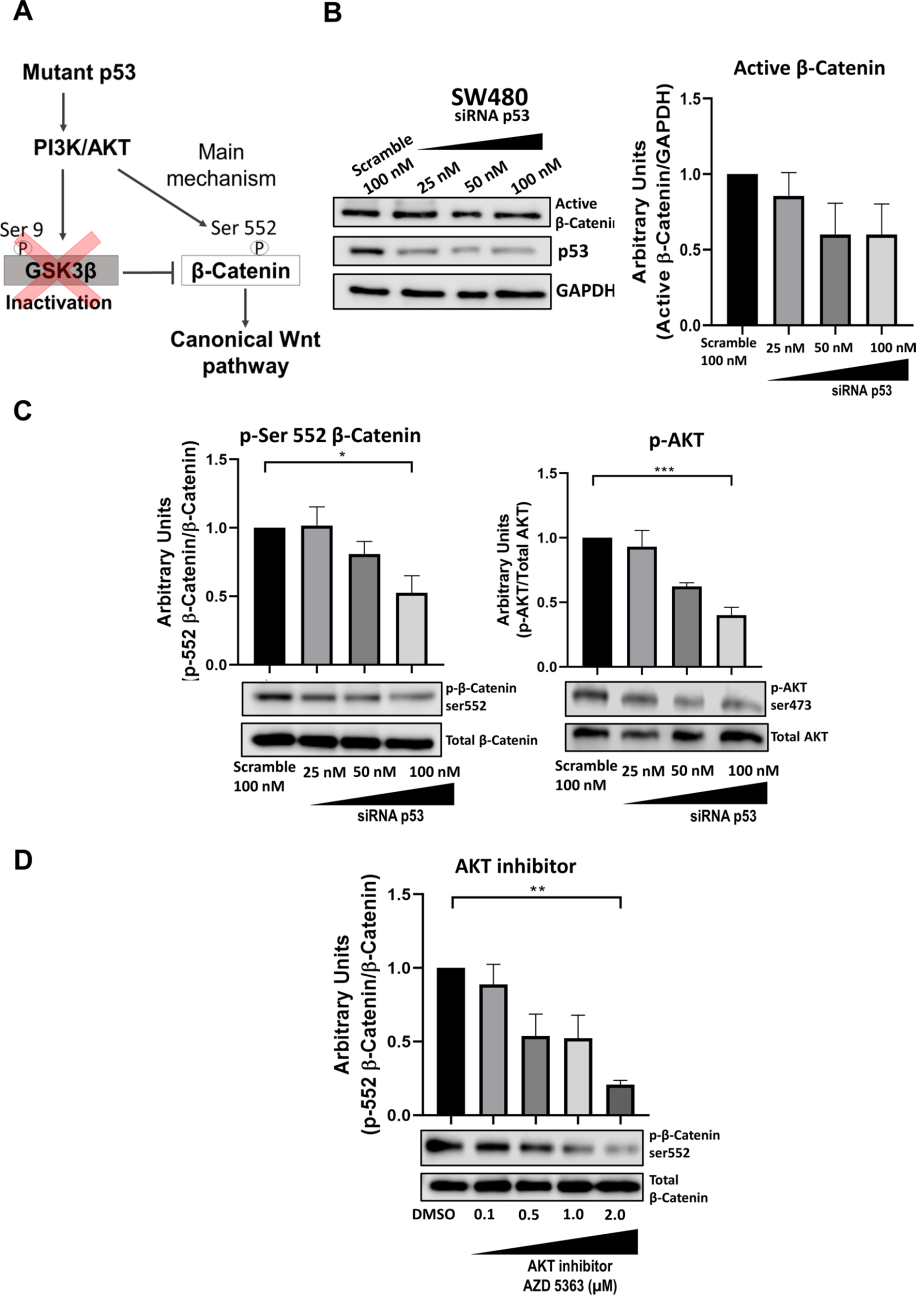
Mutant p53 knockdown decrease p-β-catenin Ser552 in a specific manner of AKT axis. **A** Schematic representation of the mechanisms influencing β-catenin activity in colon cancer cells. **B** Representative immunoblot and graph of non-phosho β-Catenin (active); GAPDH was used as a loading control. **C** Representative immunoblots and graphs of p-β-catenin Ser552 normalized to total β-catenin and p-AKT Ser473 normalized to total AKT. **D** Representative immunoblot and graph of p-β-catenin Ser552 normalized to total β-catenin measured in SW480 cancer cells treated with different doses of AKT inhibitor (AZD-5363) in the range from 0.1 to 2.0 μM; DMSO was used as control. Densitometric analysis from at least three independent experiments is shown in graphs, (means ± SEM). *p < 0.05, **p < 0.01, ***p < 0.001

To explore if mut-p53 GOF in colon SW480 cells activate Akt to induce the phosphorylation of β-catenin at Ser 552 (depicted in Fig. 4A), we first corroborated that the knockdown of mut-p53 in SW480 cells does not affect the active β-catenin levels in these cells. Indeed, as shown in Fig. 4B, the levels of active β-catenin were not significantly modified by mut-p53 knockdown. However, mut-p53 silencing decreased p-β-catenin Ser552 and Akt activation, visualized as a decrease in p-Akt Ser 473, in a dose-dependent manner of siRNA (Fig. 4C). To confirm that Akt produces β-catenin Ser 552 phosphorylation, we made use of the Akt-specific inhibitor AZD-5363. The results presented in Fig. 4D clearly show that β-catenin Ser 552 phosphorylation was diminished in a dose-dependent manner by the Akt-specific inhibitor.

Altogether, these results suggest that the mechanism by which mutant p53 stimulates canonical Wnt activation in colon cancer cells with a nonfunctional β-catenin degradation complex is by Akt-mediated phosphorylation of β-catenin at Ser 552.

### Mut-p53 participates in the induction of chemoresistance to 5-FU treatment in colon cancer cells

**Fig. 5:**
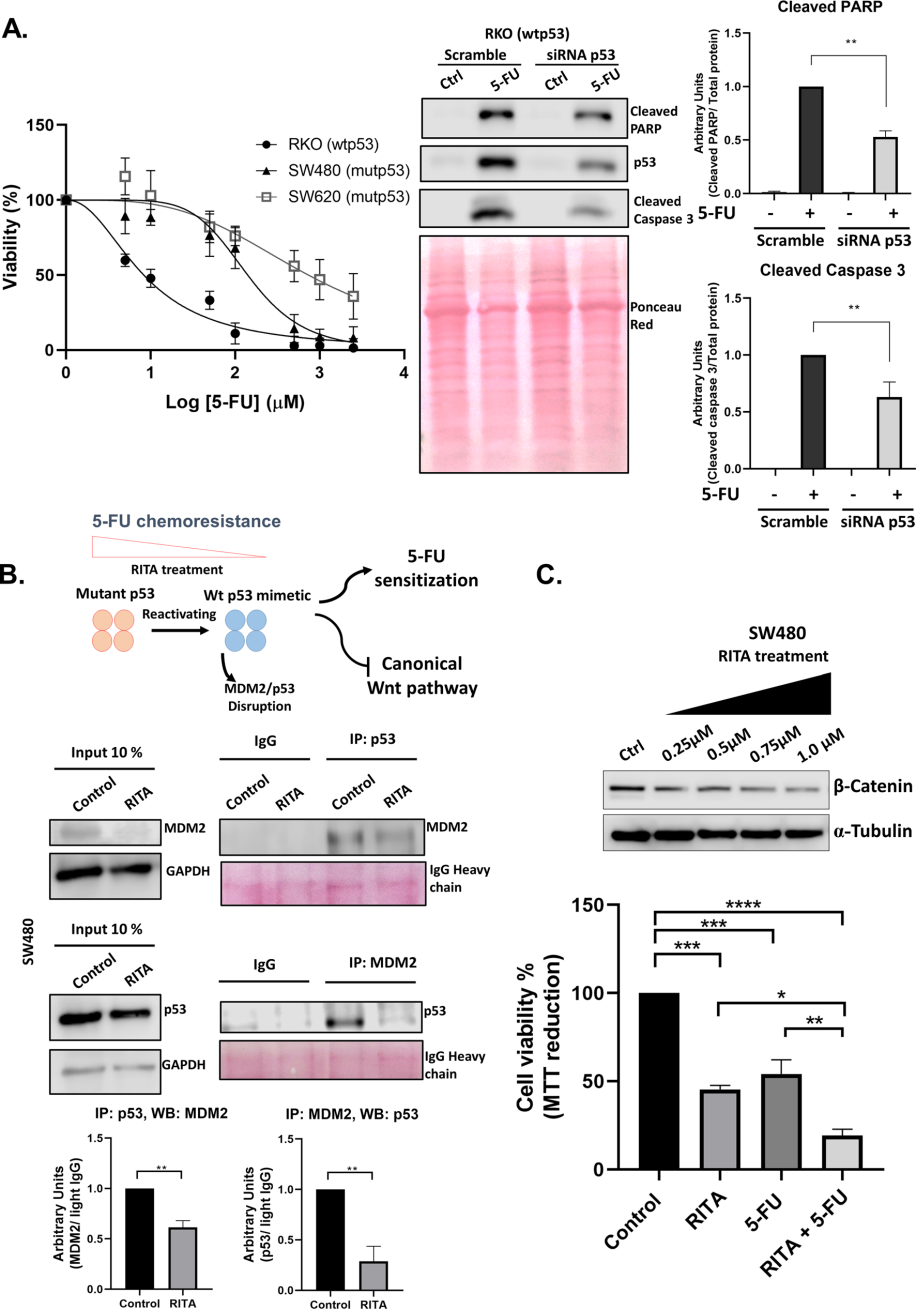
The functionality of p53 can determine the cytotoxic effect of 5-FU. **A** Dose–response curve of the RKO, SW480, and SW620 cell lines treated with 5-FU for 72 h; IC50 values were calculated on the basis of cell viability determined by MTT assay. Estimated IC50 values were RKO = 10.79 μM, SW480 = 149.6 μM, and SW620 = 817.1 uM. Representative immunoblot and graphs of cleaved caspase 3 and cleaved PARP in the RKO cell line transfected with scramble or p53 siRNA for 24 h and then treated with 5-FU at the IC50 value. Densitometric analysis was performed using total protein with ponceau staining. **B** Immunoprecipitation assay (IP) for MDM2 or p53 were measured in the cellular lysates of SW480 cell line treated for 72 h with DMSO or RITA at 1 μM. Western blot analysis for each antibody was performed and input lysate 10% was considered as control; arbitrary units were determined considering the values of IgG light chain. **C** Cell viability assay of the SW480 cell line co-treated with 5-FU in curve response doses and RITA at 1 μM for 72 h. Graphs represent the analysis from at least three independent experiments, (means ± SEM). *p < 0.05, **p < 0.01, ***p < 0.001

We recently reported that canonical Wnt activation plays an essential role in inducing chemoresistance to 5-Fluorouracil (5-FU) in colon cancer cells (Moreno-Londoño et al. 2023). Given that in this study, we found that mut-p53 activates β-catenin/Wnt signaling, we then investigated whether mut-p53 could also participate in this resistance induction. First, we measured the effect of increasing concentrations of 5-FU for 72 h on cell viability using RKO cells expressing wt-p53 and SW480 or SW620 cells expressing mut-p53. In agreement with our previous reports, we observed that RKO cells are more sensitive to 5-FU (IC-50 value of 10.79 μM) than SW480 or SW620 cells, which are highly resistant to 5-FU (149.6 and 817.1 μM respectively) (Fig. 5A). Interestingly, when we blocked wt-p53 expression in RKO cells, 5-FU induced cell death, visualized as cleaved-PARP (c-PARP) and cleaved Caspase 3 apoptosis markers, was significantly diminished, as can be observed in Fig. 5A.

When we tried to investigate the effect of transient knockdown of mut-p53 on 5-FU exposure of SW480 cells, we noticed that it is very difficult to efficiently obtain a decrease in the amount of mut-p53 protein levels in these cells (data not shown). Thus, we decided to measure the effect of reestablishing canonical functions of wt-p53 in SW480 cells employing the small-molecule RITA (Reactivation of p53 and Induction of Tumor cell Apoptosis) (Grinkevich et al. 2009; Zhao et al. 2010), as depicted in Fig. 5B. It has been described that RITA can reactivate mut-p53 to induce its canonical tumor suppressor functions. Although RITA mechanism action is not clearly defined, it has been reported that the main mechanisms by which RITA acts is by disrupting the interaction of the MDM2/p53 complex. We validated this effect of RITA in mut-p53-expressing SW480 cells employing a reciprocal co-immunoprecipitation assay. Figure 5B shows how RITA treatment produced a significant decrease in the MDM2/p53 interaction (Fig. 5B) and importantly, that RITA treatment re-established the negative effect on active β-catenin levels in a dose-dependent manner, mimicking the action of wild-type p53 on β-catenin levels (Fig. 5C). Finally, we analyzed the effect of both RITA and 5-FU treatment on cellular viability of SW480 cancer cells. As can be seen in Fig. 5C, RITA treatment alone decreased the cellular viability; however, the combination of 5-FU and RITA produced an additive effect, making SW480 more sensitive to 5-FU treatment (Fig. 5C). Taken together, our results indicate that mut-p53 expression in colon cancer cells favors the induction of chemoresistance to 5-FU and suggest that it may be by stimulating the canonical Wnt pathway in colon malignant cells.

## Discussion

The Wnt and p53 pathways play essential roles in cancer initiation and progression as they act as central hubs of many cancer hallmarks (Xiao et al. 2022). The Wnt signaling pathway is a key regulator of intestinal epithelium homeostasis and is aberrantly activated in most colon cancer cases (Clevers and Nusse 2012).

The p53 protein acts as a major regulator in many cell-fate-determining processes and as a sensor for a variety of cellular stress signals, including DNA damage, oncogene activation, and hypoxia (Kim and Lozano 2018). It is found to be mutated in nearly 50% of human cancers, including colon cancer. Aside from abolishing the tumor-suppressive capacities of the wild-type form, p53 mutations confer novel oncogenic functions that impact molecules and pathways, a phenomenon termed mutant p53 gain-of-function (GOF) (Kim and Lozano 2018; Nakayama et al. 2020). Among these mutations are the hot spots Arg175His, Arg249Ser, and Arg273His. SW480 colon cancer cells express mut-p53-R273H, a DNA-contact mutant unable to specifically bind DNA (Prowald et al. 2007). Even though mut-p53 GOF has not been completely defined, essential mechanisms related to malignant progression have been described in critical functions such as proliferation, metastasis, metabolic disbalance, immune evasion, and autophagy. Moreover, *TP53* status constitutes an important criterion for defining the chemotherapeutic effect of pharmacological treatments (Alvarado-Ortiz et al. 2021; Cordani et al. 2016).

Accumulated experimental evidence has demonstrated that p53 and Wnt signaling pathways cooperate with each other to drive cancer initiation and progression (Muller and Vousden 2013; Xiao et al. 2022). Besides, current cancer genomics data analysis has indicated a cancer type-specific interplay between mutations in the Wnt and p53 pathways. Consistent with this, we performed an in silico analysis using the TCGA and GEPIA public databases and found that high RNA-seq expression of mutant p53 in colon adenocarcinoma samples correlates with high expression of canonical Wnt elements, such as β-catenin, as well as c-Myc, a typical canonical Wnt gene target.

One of the first demonstrations of mutant p53 GOF was observed using null-*TP53* cells that were transfected with the mutant p53 over-expression vector and injected into mice; it was observed that they formed lethal tumors in mice compared with control null-*TP53* cells (Brosh and Rotter 2009; Stein et al. 2019)*.* Here, we made use of null-*TP53* H1299 cells transfected with wt-p53 or mut-p53 to compare their effects on endogenous β-catenin protein levels. We found that only wt-p53, but not its mutants, diminished β-catenin levels in a dose-dependent manner and expressed p21, a typical wt-p53 gene target. In agreement with our results, it has been reported that p53 inhibits canonical Wnt- β-catenin signaling in different tumor cell types (Kim et al. 2011; Prowald et al. 2007). The underlying mechanism relies on ubiquitination and proteasomal degradation of β-catenin, which requires the function of GSK-3β, a core component of the β-catenin destruction complex. Inhibition of this enzyme thus increases β-catenin levels. However, Prowald et al. (2007) reported that the expression of both wt-p53 and mut-p53-His273 in prostate cancer cells PC3 were able to reduce the protein levels of an exogenously expressed degradation-resistant form of β-catenin, implying that functional GSK-3β is not necessarily required for downregulation of β-catenin in these cells, indicating that the interactions between p53 and the β-catenin destruction complex are highly diverse (Prowald et al. 2007; Xiao et al. 2022). In addition, Kim et al. (2011), showed that wt-p53 negatively regulates some transcripts related to the canonical Wnt pathway, like Wnt1a, Wnt3a, LRP6, β-catenin, and LEF1. This effect seems to be dependent on miR34-a, since this microRNA is positively increased by wt-p53.

Interestingly, in this study, we also found that the β-catenin levels were not increased as a result of mut-p53-R273H expression in null-*TP53* H12999 cells, with respect to the levels found in nontransfected or empty controls (Fig. 2A, B). In contrast with these results, Cagatay and Ozturk (2002) reported that in hepatocellular carcinoma cells, mut-p53 increases β-catenin levels, possibly through regulation of Siah1 (Seven in absentia homolog 1), a target of wt-p53 that participates in β-catenin degradation. Thus, while it is well known that wild-type p53 acts negatively in several ways in regulating canonical Wnt signaling, so far, much less is known about the mechanisms of how mut-p53 could drive tumor cell invasion and activate Wnt/β-catenin signaling. Previous reports have also shown that mut-p53 favors tumorigenic capacities and induces the expression of stem cell markers, such as ALDH1A1, CD44, or Lgr5, in colorectal cancer cells, suggesting an important effect of GOF in this type of disease (Solomon et al. 2018).

The colon cancer cells SW480 or SW620 employed in this study expressing mut-p53 display constitutive active canonical Wnt signaling because they express a truncated APC protein and thus do not have a functional β-catenin degradation complex. We reasoned that the inhibition of GSK-3β by its phosphorylation at Ser 9, observed in null-*TP53* H1299 cell line expressing mut-p53, would not affect the β-catenin levels in SW480 cells, and thus, that there would exist another mechanism in these cells to explain how mutant p53 can stimulate β-catenin-mediated transcriptional activity. The most salient finding obtained in this work was the demonstration that mut-p53-R273H expressed in SW480 colon cancer cells stimulates canonical Wnt/β-catenin signaling by activating the PI3K/ AKT pathway. Specifically, we found that mut-p53 activates Akt, inducing the phosphorylation of β-catenin at its Ser552, which contributes to β-catenin stability and enhances its transcriptional activity (Behrouj et al. 2021; Fang et al. 2007; Wang et al. 2020). Consistent with this, experimental evidence has shown that mut-p53 GOF can increase pathways like PI3K/AKT, small GTPases (Ras, Rho, Rac1), and the activity of transcription factors related to malignant processes, such as HIF1α or NF-kB (Alvarado-Ortiz et al. 2021). In this regard, Valentino et al., in 2017, reported that several effects in breast and prostate cancer attributed to mut-p53 are mediated by regulation of the PI3K/AKT pathway. This occurs through the binding of mut-p53 with DAB21P, a scaffold protein that binds to and inactivates p85-PI3K, impairing its repressive functions on PI3K and promoting the intracellular effects of AKT (Valentino et al. 2017). Also, in agreement with our findings, Huang et al. 2021, reported that knockdown of mutant p53 suppressed the proliferation and migration of MDA-MB-468 cells by inhibiting the PI3K/AKT**/**mTOR signaling pathway. And interestingly, Chen M et al., showed in 2022 that genotoxic stress induces nuclear Akt activation via a p53-dependent mechanism in several cancer cell lines (Chen et al. 2022).

Given that in this study we found that mut-p53 activates β-catenin/Wnt signaling, and we have recently reported that canonical Wnt activation plays an essential role in inducing chemoresistance to 5-FU in colon cancer cells (Moreno-Londoño et al. 2023), we investigated whether mut-p53 could participate in the induction of chemoresistance to 5-FU. We found that endogenous expression of wtp53 explains the sensitivity to low doses in the RKO cell line, contrary to mutp53, where more concentration of 5-FU is needed to induce the same effect. In agreement with this notion, previous reports reveal that colon cancer cells expressing wtp53 are sensitive to 5-FU, meanwhile those resistant to 5-FU show higher canonical Wnt signaling and less activity of p53 (He et al. 2018; Xie et al. 2017). Although the impact of mutp53 on 5-FU resistance in colon cancer has been widely reported (Cho et al. 2020; Huang et al. 2019), the mechanisms through which such resistance is achieved are not clear. In recent years, some small-molecular compounds have been investigated intensively for their ability to reactivate and restore p53 via different mechanisms. We made use of RITA (Reactivation of p53 and Induction of Tumor cell Apoptosis). This compound specifically targets mutant oncogenic p53 protein, restoring the sequence-specific DNA binding region and activating several p53 target genes. This compound directly binds to p53 rather than MDM2 and induces a conformational change in p53, which interferes with the p53-MDM2 interaction, and p53 ubiquitination, resulting in its activation (Grinkevich et al. 2009; Zhao et al. 2010)*.* We showed here that RITA treatment of SW480 cells re-established the negative effect on β-catenin levels in a dose-dependent manner, mimicking the action of wild-type p53 on β-catenin levels (Fig. 5C). These results, therefore, suggest that re-establishing the canonical functions of p53 can overcome chemoresistance to 5-FU. Notably, our results indicate that mut-p53 expression in colon cancer cells favors the induction of chemoresistance to 5-FU by stimulating the canonical Wnt/β-catenin pathway.
